# Thoracoscopic esophagectomy for esophageal carcinoma after peroral endoscopic myotomy for esophageal achalasia: a case report

**DOI:** 10.1186/s40792-021-01270-z

**Published:** 2021-08-19

**Authors:** Junichi Tsunokake, Yusuke Taniyama, Fumiyoshi Fujishima, Chiaki Sato, Hiroshi Okamoto, Toshiaki Fukutomi, Yohei Ozawa, Naoto Ujiie, Ken Koseki, Yusuke Gokon, Makoto Horiuchi, Ryujiro Akaishi, Takuro Yamauchi, Michiaki Unno, Takashi Kamei

**Affiliations:** 1 Department of Surgery, Tohoku University Graduate School of Medicine, 1-1 Seiryo-machi, Aoba-ku, Sendai, Miyagi 980-8574 Japan; 2 Department of Pathology, Tohoku University Hospital, 1-1 Seiryo-machi, Aoba-ku, Sendai, Miyagi 980-8574 Japan

**Keywords:** Esophageal achalasia, Peroral endoscopic myotomy (POEM), Thoracoscopic esophagectomy

## Abstract

**Background:**

Esophageal achalasia causes dysphagia following impaired relaxation of the lower esophageal sphincter due to the degeneration of Auerbach’s plexus in the esophageal smooth muscle. Recently, peroral endoscopic myotomy (POEM) has become one of the preferred treatment options for esophageal achalasia. However, pathomorphological changes after POEM have not been well examined.

**Case presentation:**

A 65-year-old man with a history of POEM for esophageal achalasia was diagnosed with clinical stage II (cT2-N0-M0) thoracic esophageal squamous cell carcinoma and was consequently treated with neoadjuvant chemotherapy followed by thoracoscopic esophagectomy. Intraoperatively, the esophagus appeared dilated, reflecting esophageal achalasia; however, fairly slight fibrous adhesions were observed between the esophagus and the pericardial surface despite previously performed POEM via an anterior incision. Histopathological examination revealed esophageal wall thickening, edema, and fibrosis extending from the lamina propria to the submucosa. Besides, the majority of the inner layer and some proportion of the outer layer of the muscularis propria were found to be missing or atrophic at the esophagogastric junction (EGJ). No ganglion cells could be detected at the Auerbach’s plexus.

**Conclusions:**

The previous history of POEM did not affect circumferential mediastinal periesophageal dissection during thoracoscopic esophagectomy. Nevertheless, a large proportion of the inner layer of the muscularis propria at the EGJ level seemed to have become lost or atrophic because of the POEM procedure.

## Background

Esophageal achalasia, which was first reported in 1697 as idiopathic esophageal dilatation, manifests as dysphagia and is characterized by the strong contraction of the lower esophageal sphincter (LES) and its failure to relax due to the degeneration of Auerbach’s plexus in esophageal smooth muscle [1]. It also involves the risk of esophageal squamous cell carcinoma (ESCC) even after treatment [2]. Therefore, clinicians should follow-up patients with esophageal achalasia for a careful long-term surveillance. Although there are currently no radical treatments to cure esophageal achalasia, the Heller–Dor surgery (or balloon dilatation) represents the standard treatment to relieve the symptoms [3]. Recently, peroral endoscopic myotomy (POEM), which can be safely performed in an endoscopic unit, has also been introduced [4]. Briefly, it entails creating a submucosal tunnel from the proximal end of the pathological contraction all the way past the esophagogastric junction (EGJ), performing myotomy of the inner circular muscle of the muscularis propria (including the LES complex), and closing the mucosal entry with clip placement. This new technique is less invasive and thus globally attempted for esophageal achalasia treatment. However, pathomorphological changes of the esophagus after POEM have not been studied so far. Here, we present a case of middle thoracic ESCC in a patient with a history of POEM for esophageal achalasia from whom tissue specimens were obtained to evaluate post-POEM changes in esophageal morphology.

## Case presentation

A 65-year-old man visited our hospital for follow-up esophagogastroduodenoscopy (EGD) after four times undergoing endoscopic submucosal dissection (ESD) for early esophageal cancer. He had no habit of drinking, but had a history of smoking (20 cigarettes per day for 40 years) until POEM. He also had a past history of esophageal achalasia classified as type II according to the Chicago classification system (straight type, grade II), for which he had received POEM 6 years earlier as follows: mucosal incision creation at 2 o’clock position, 32 cm from an incisor, followed by anterograde submucosal tunneling and myotomy at a distance of 34–50 cm from the incisor, with the EGJ being 48 cm from the incisor. His symptoms markedly improved after POEM, and his Eckardt score improved to 0. He underwent follow-up EGD every 6 months after POEM and ESD.

**Fig. 1:**
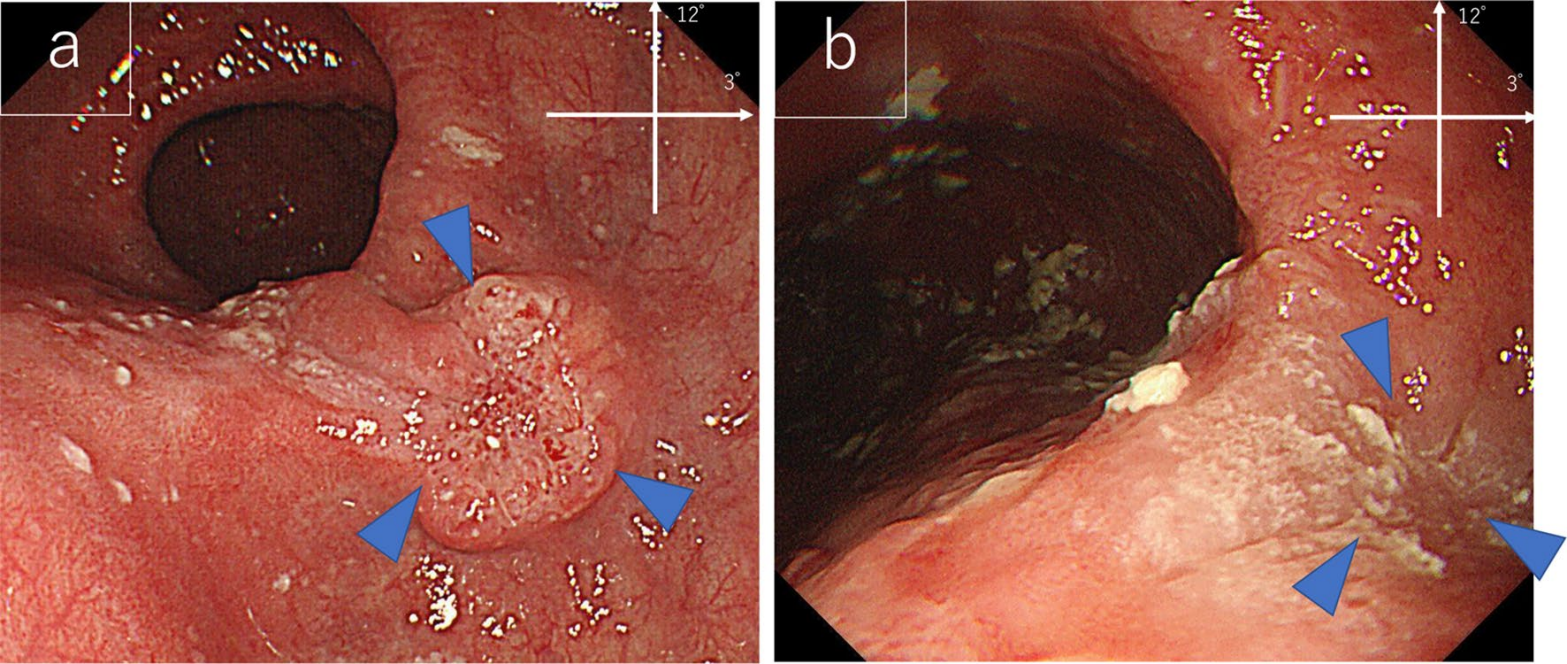
Esophagogastroduodenoscopy of the middle thoracic esophageal lesion (arrow head), **a** before neoadjuvant chemotherapy and **b** immediately before surgery

**Fig. 2:**
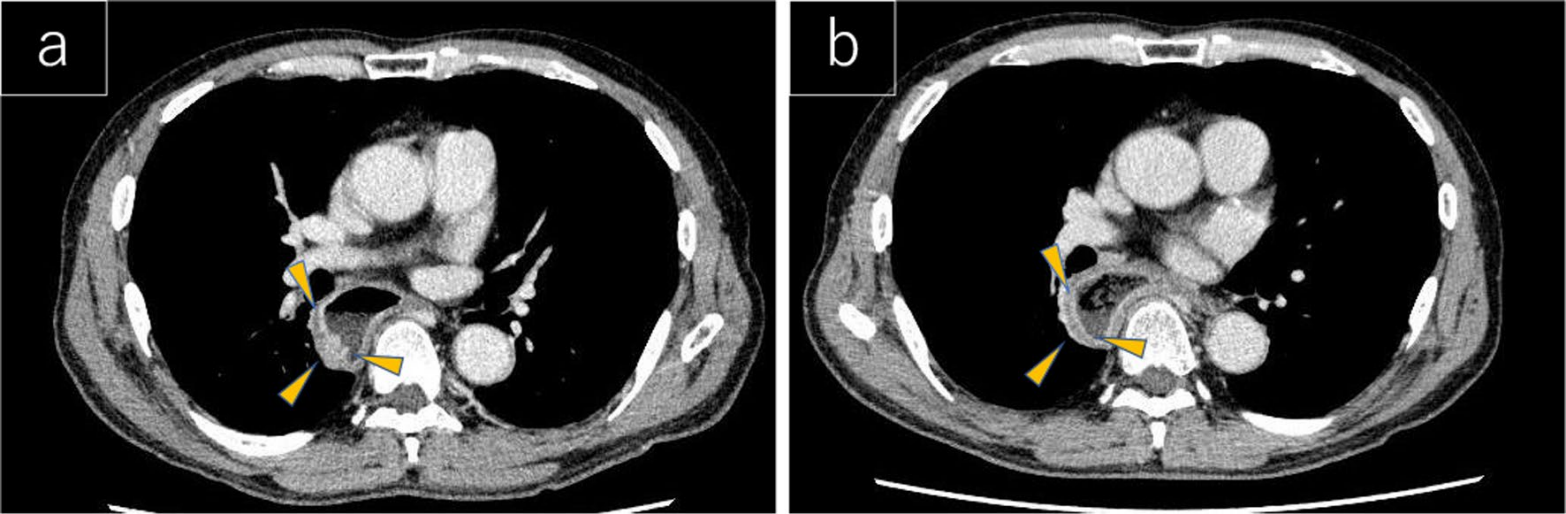
Computed tomography images showing middle thoracic esophageal wall thickness (arrow head), **a** before neoadjuvant chemotherapy and **b** immediately before surgery

**Fig. 3:**
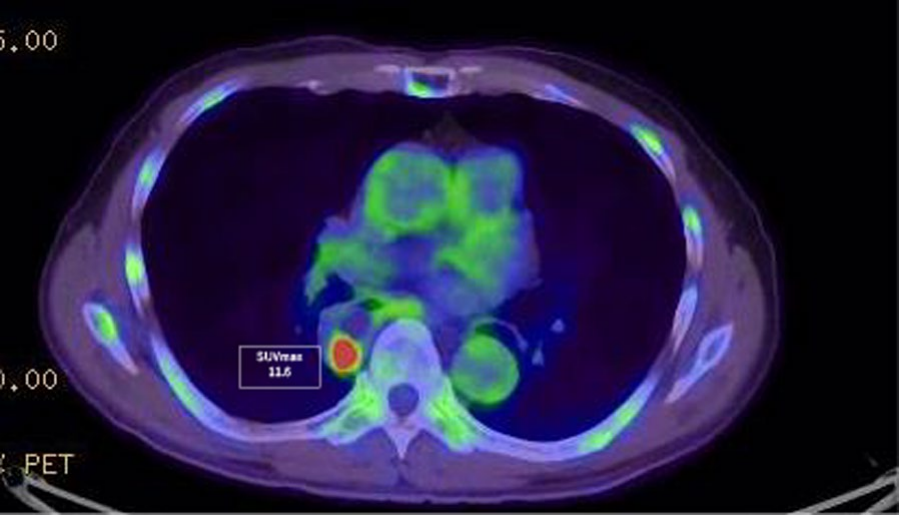
Positron emission tomography–computed tomography image showing fluorodeoxyglucose accumulation in the middle thoracic esophageal wall

**Fig. 4:**
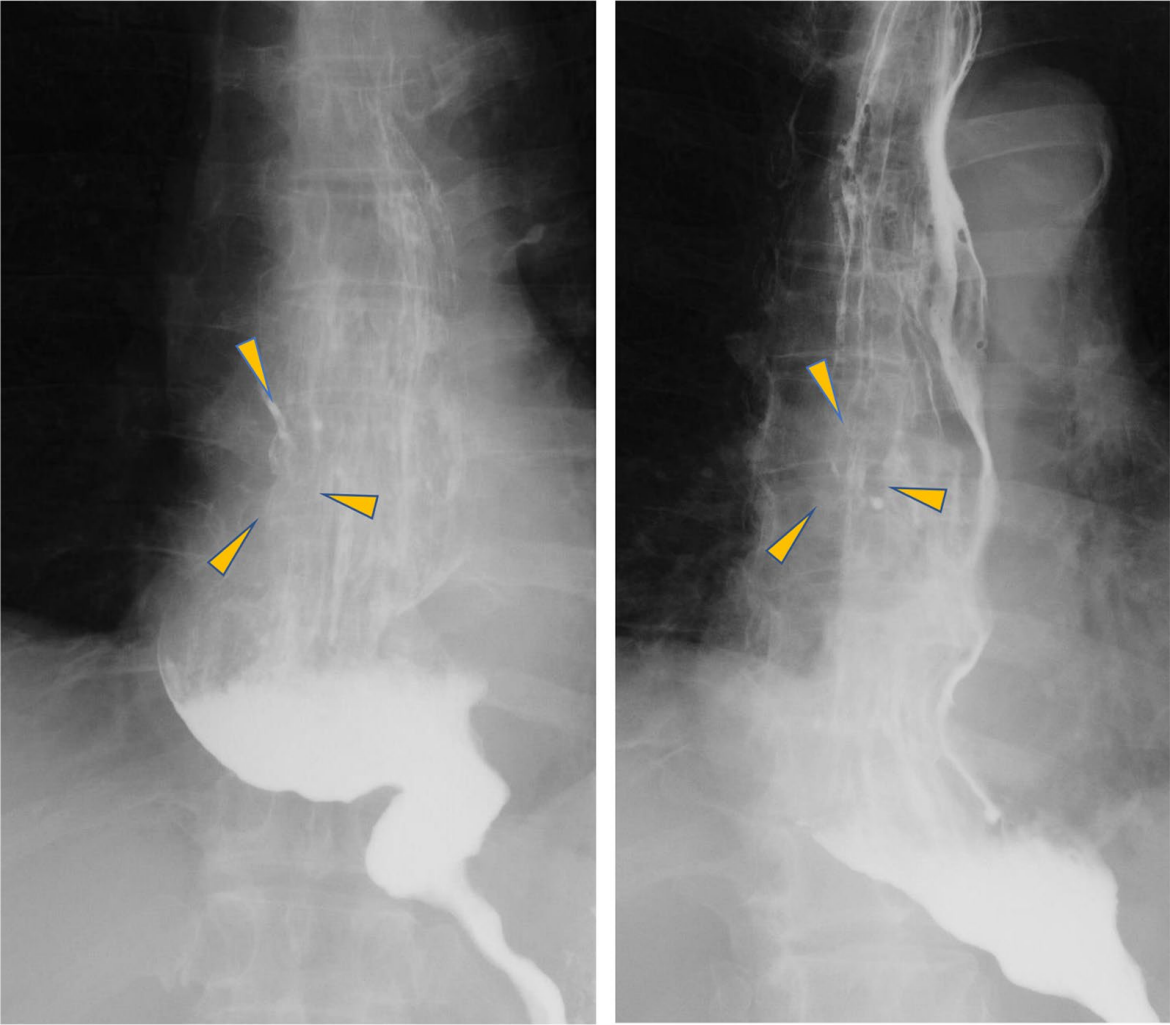
Preoperative fluoroscopy with gastrografin (arrow head, tumor). Although the esophagus on the anal side from Mt to the EGJ was dilated, passage of gastrografin is smooth

**Fig. 5:**
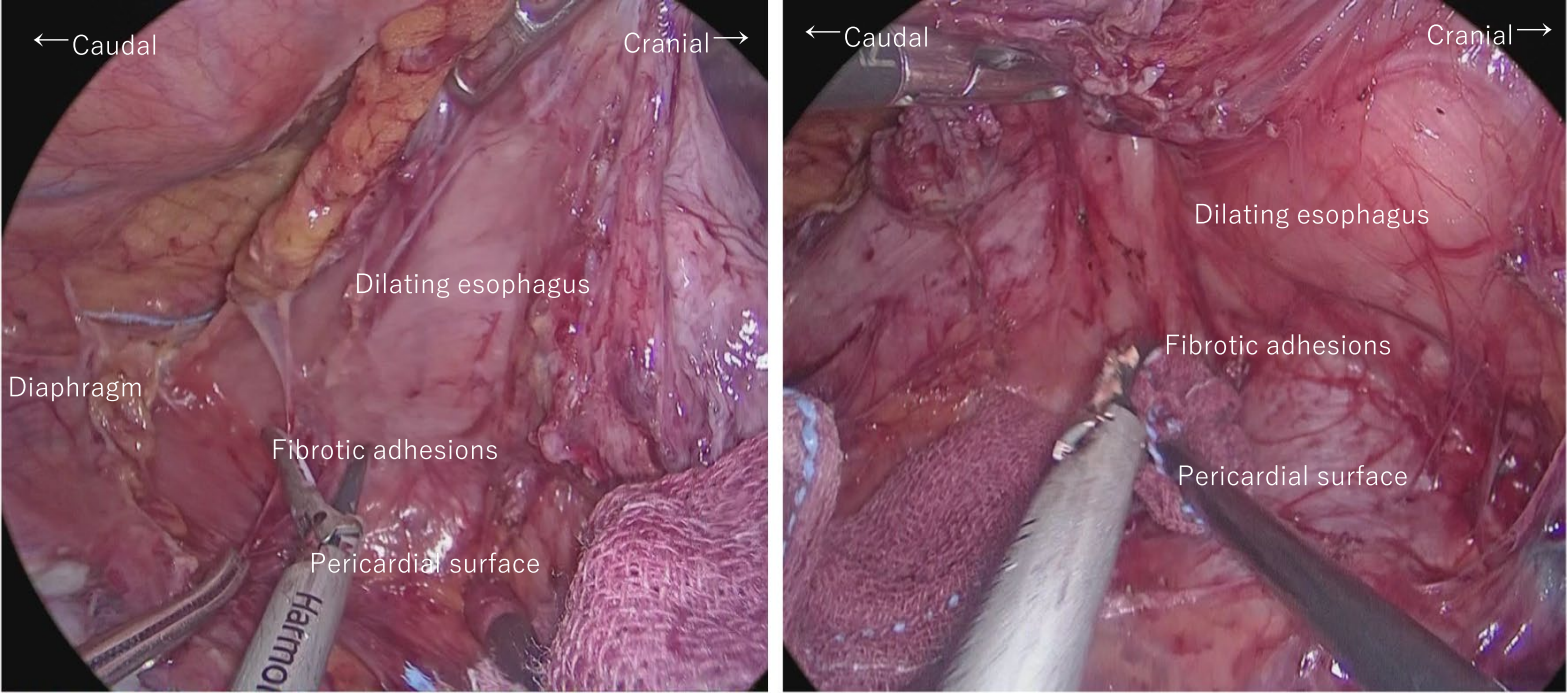
Thoracoscopic procedure. There was marked esophageal dilatation due to achalasia. Although the dilated esophagus required intervention, the procedure could be performed safely without any major intraoperative issues

**Fig. 6:**
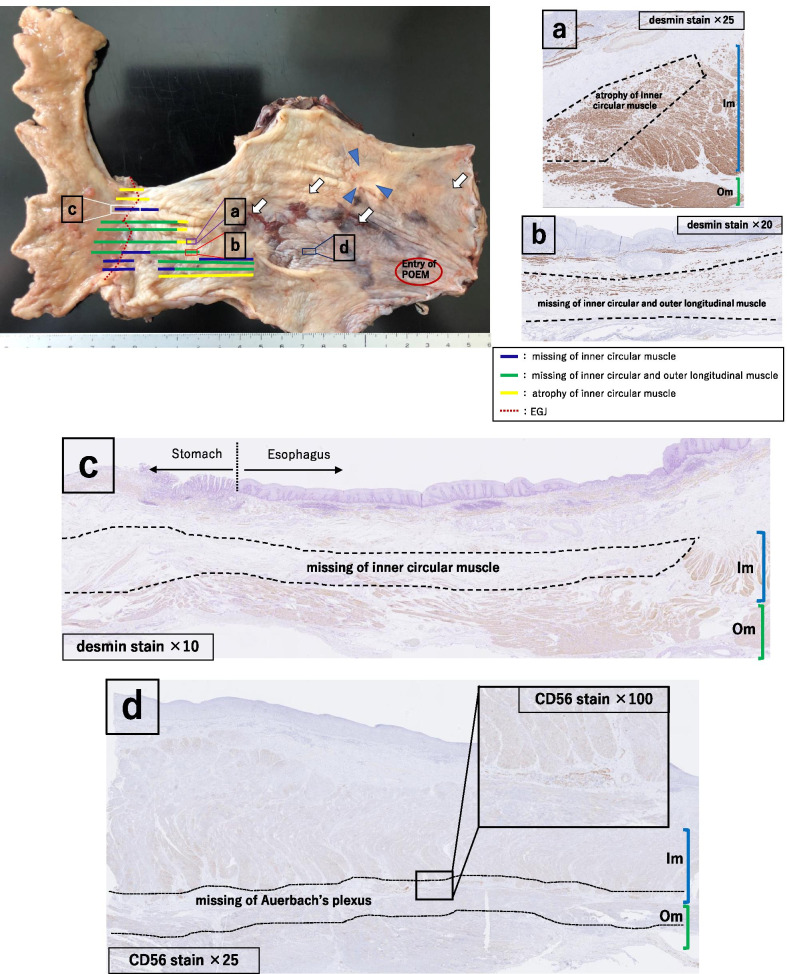
Excised specimen (left section). The esophagus is dilated, and the mucosal surface is rough. Arrowheads indicate the lesion and arrows indicate the endoscopic submucosal dissection scars. Histopathological findings (**a**–**d**): **a** atrophy of inner circular muscle (desmin staining; magnification, ×25), **b** missing inner circular and outer longitudinal muscle (desmin staining; magnification, ×20) *Im: inner circular muscle, Om: outer longitudinal muscle. Note the loss and atrophy of muscularis propria extending from the esophagogastric junction to the oral side, **c** missing inner circular muscle in the stomach area (desmin staining; magnification, ×10), **d** missing ganglion cells in Auerbach’s plexus (CD56 staining; magnification, ×25, ×100) *Im: inner circular muscle, Om: outer longitudinal muscle

EGD examination revealed a tumorous lesion in the middle thoracic esophagus at 4–5 o’clock position (Fig. 1a, b). Endoscopic biopsy specimens from this lesion, which had never been treated with ESD were indicative of squamous cell carcinoma. Computed tomography showed slight wall thickening in the middle esophagus without lymph node swelling (Fig. 2a, b), and positron emission tomography demonstrated high accumulation of fluorodeoxyglucose with a maximum standardized uptake value of 11.6 at the tumor lesion (Fig. 3). Fluoroscopy with gastrografin revealed a cancer site and smooth passage of gastrografin around the EGJ despite the dilatation of the esophagus (Fig. 4). On the basis of these findings, we reached the clinical diagnosis of cT2-N0-M0 (stage II) ESCC according to the tumor-node-metastasis (TNM) classification (eighth edition). Thus, neoadjuvant chemotherapy with fluorouracil and cisplatin was initiated for the patient. Five weeks after the end of the chemotherapy, thoracoscopic esophagectomy was performed via the right thoracic approach in the prone position under artificial pneumothorax induced by carbon dioxide insufflation at a pressure of 6–8 mmHg. During surgery, the esophagus was found to be dilated which suggested esophageal achalasia. However, there were very slight fibrous adhesions between the esophagus and the pericardial surface despite the POEM procedure formerly conducted through an anterior incision (Fig. 5). A hand-assisted laparoscopic method was applied for the abdominal procedure, and reconstruction was conducted with a gastric tube positioned in the posterior mediastinal route. The total operation time and total intraoperative blood loss were 570 min and 113 g, retrospectively. There was no severe postoperative complication, and the patient was discharged on postoperative day 11. The pathological TNM stage of the tumor was postoperatively identified as ypT2-N0-M0 (stage IIA). Gross examination of the surgical specimen revealed that the esophagus was extremely dilated and that the mucosal surface of the esophagus was visually rough with scars of ESD (Fig. 6, left section). The histopathological analysis of the surgical specimen indicated esophageal wall thickening along with edema and fibrosis extending from the lamina propria to the submucosa. Moreover, three quarters of the inner layer and some proportion of the outer layer of the muscularis propria were missing, with some degree of atrophy present in the inner circular muscle layer at the EGJ level (Fig. 6a, b). Moreover, the inner circular muscle of stomach was sufficiently incised (Fig. 6c). Ganglion cells could not be detected in the normal location of the Auerbach’s plexus (Fig. 6d).

## Discussion

This is a rare case report of thoracoscopic esophagectomy for ESCC after POEM for esophageal achalasia. The first report on the relationship between ESCC and esophageal achalasia was published in 1872 [5]. The incidence of esophageal achalasia estimated to be 0.5–1.6 cases in 100,000 individuals per year [6, 7], with 0.4–9.2% of all cases suffering from ESCC [8]. Esophageal achalasia was mainly managed with pneumatic dilation, and Heller myotomy (HM) before POEM, which entails incision of the inner circular muscle through a submucosal tunnel around the EGJ, was introduced by Inoue in 2009 as a novel and effective treatment option with less invasiveness [4]. Recent studies have compared POEM with other methods for the treatment of esophageal achalasia. Steven G and Fraukje A have reported that although POEM is associated with a high incidence of reflux esophagitis, which can be controlled by proton pump inhibitors, it is superior to pneumatic dilation and HM in terms of long-term therapeutic effects as evaluated by the Eckardt score [9, 10]. Therefore, POEM is now becoming one of the most common standard procedures to treat esophageal achalasia.

In the present case, the pathological assessment of the surgical specimen revealed that nearby 75% of the inner circular muscle was lost at the EGJ level, which extended along the 2 o’clock direction toward the oral side of the entry hole. As shown in Fig. 6, the loss in the outer longitudinal muscle, as well as atrophied inner circular muscle, could also be detected along this area. The observed loss and atrophy resulted from the muscular incision made during POEM to relieve the LES pressure and improve the esophageal passage.

In general, only the inner circular muscle is incised when the EGJ is approached through the mucosal entry in the course of POEM. Although the smooth muscles have the ability to regenerate [11], there was nearby 75% muscle loss in addition to the presence of some atrophic areas in the inner circular muscle at the EGJ level; these changes were more extensive than the incised site by POEM. Barry and Aymeric reported that muscle incisions caused muscular atrophy along the fiber direction in the rabbit gastrocnemius [12, 13]. This is because the rupture of the strong tension caused by esophageal achalasia at the EGJ leads to remarkable atrophy along the fiber direction of the inner circular muscle. The esophageal inner circular muscle was missing in tiers in some area, which might have caused by the bending and dilatation of esophagus from the cranial to the caudal side; this phenomenon might hinder the incision of the esophageal inner circular muscle in a straight line. This outcome might also have been caused by the discrepancy between the incisional line and the longitudinal axis of the esophagus during specimen incision because of the natural winding around the EGJ. There was a loss of the outer longitudinal and inner circular muscles along the submucosal tunnel. This is attributable to the decreased strength of the esophageal wall at the site of incision and the subsequent pressure that causes a tear in the outer longitudinal muscle along its fiber direction.

Although we preserved the outer longitudinal muscle layer during POEM, we assumed that inflammation would extend beyond the esophageal adventitia, thereby resulting in strong adhesion formation between the surrounding tissues. However, fibrous formation around the esophagus (including the pericardial surface) was not as intense as expected; therefore, the exfoliation of the esophagus from the surrounding tissues was relatively easy. In general, some minor mediastinal emphysema may occur immediately after POEM [8]. Except for the rare cases of mediastinitis due to post-POEM mucosal injury [14], POEM has little impact outside of the esophageal adventitia.

## Conclusion

This is the first report of thoracoscopic esophagectomy performed after POEM for esophageal achalasia. The loss and atrophy of the esophageal inner circular muscle were detected in a relatively large area from the mucosal entry to the EGJ. A small proportion of the outer longitudinal muscle was also lost along the inner muscle incision area. However, the effect of POEM seems to be little beyond the esophageal adventitia, so we can safely perform thoracoscopic esophagectomy after POEM for esophageal achalasia.

## Data Availability

Not applicable.

## Abbreviations

ESCC: Esophageal squamous cell cancer
POEM: Peroral endoscopic myotomy
ESD: Endoscopic submucosal dissection
EGD: Esophagogastroduodenoscopy
NAC: Neoadjuvant chemotherapy
CT: Computed tomography
FDG: Fluorodeoxyglucose
PET: Positron emission tomography
HM: Heller myotomy
EGJ: Esophagogastric junction
LES: Lower esophageal sphincter
TNM: Tumor-node-metastasis
CD: Cluster of differentiation
